# Supplementary figures and images for: Paternally expressed gene 3 (Pw1/Peg3) promotes sexual dimorphism in metabolism and behavior
Source: PLoS Genet. 2022 Jan 13;18(1):e1010003. doi: 10.1371/journal.pgen.1010003 (PMC8791484; doi:10.1371/journal.pgen.1010003)

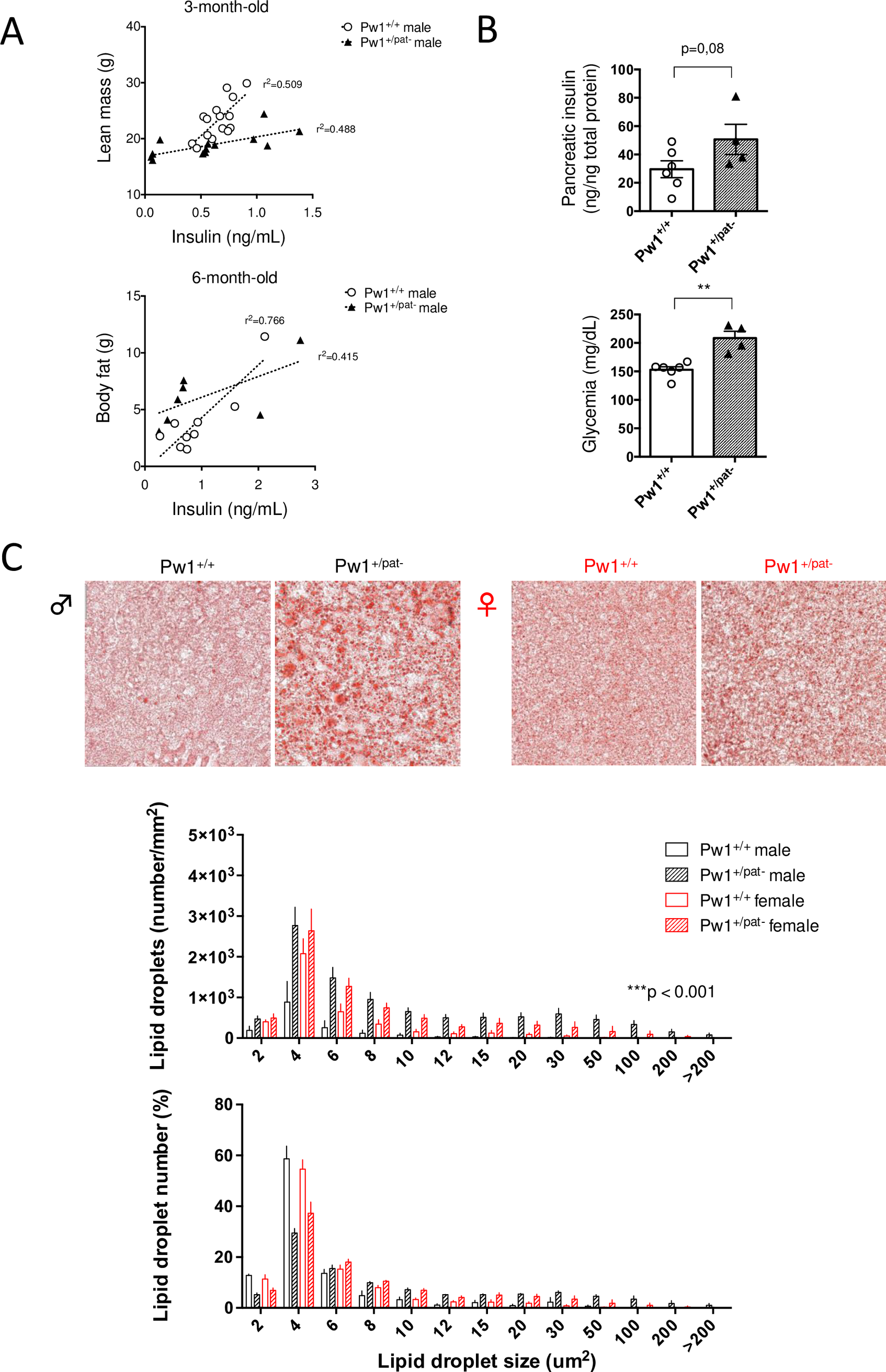

Supplement: S1 Fig — (A) Positive correlation of insulin levels with lean mass (top) and with body fat (bottom) was observed at at 3 months and 6 months of age, respectively. Correlation was determined by simple linear regression analysis. (B) Pancreatic insulin content and blood glucose levels at 10 months of age. Pancreatic insulin content was measured using acid-ethanol extraction protocol, followed by the insulin ELISA and normalized with total protein content. Columns, mean; bars, SEM; **, P< 0.01 by Mann-Whitney U test. (C) Typical images of Oil Red-O stained 8-month-old livers (top) and the quantification of lipid droplet number and size distribution (bottom), illustrating the differences in lipid content between the groups. P<0.001 by two-way ANOVA with Tukey’s multiple comparisons. Original magnifications: x200. (TIF) [file pgen.1010003.s001.tif]

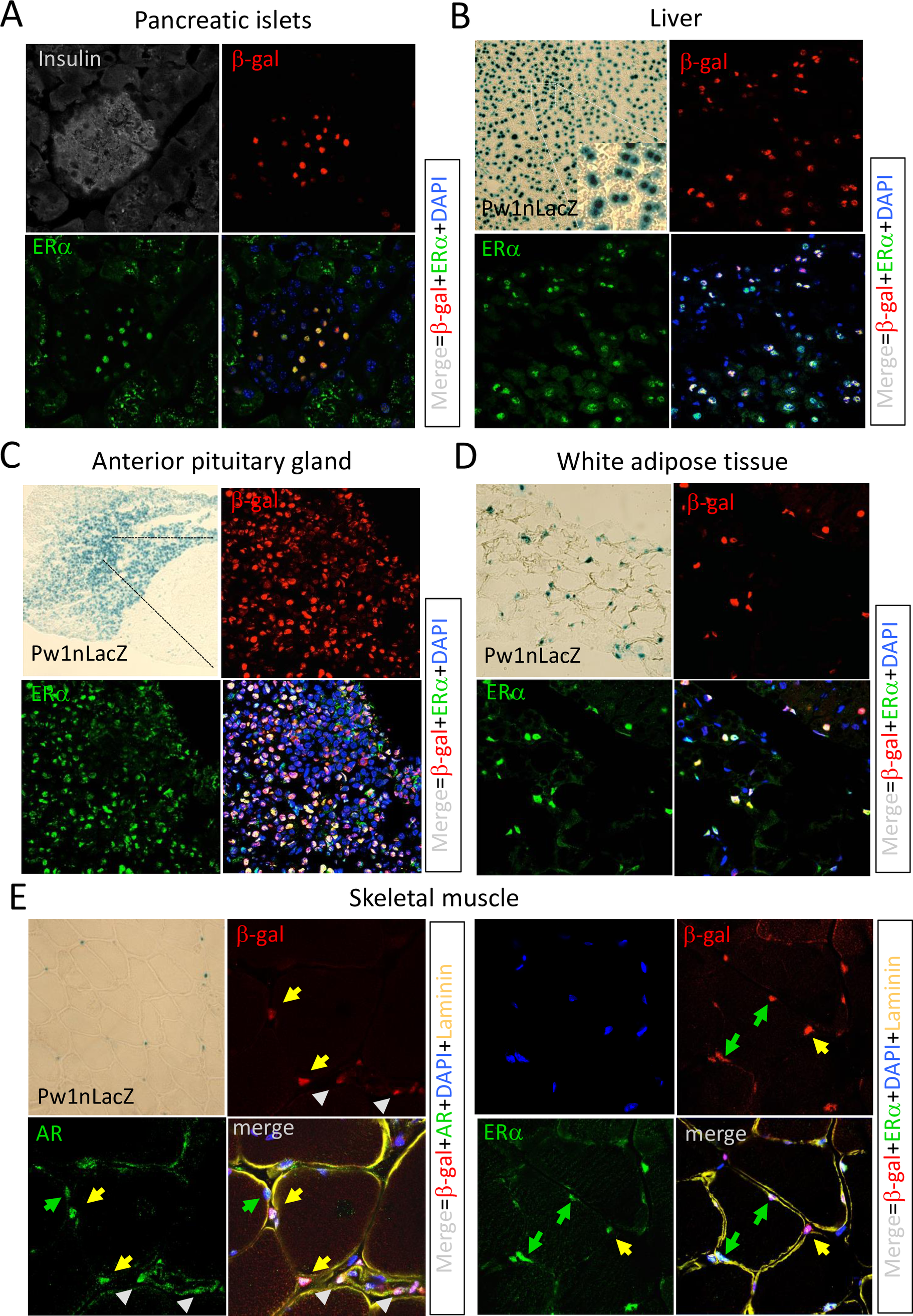

Supplement: S2 Fig — Pw1 reporter expression predominantly co-localizing with the nuclear expression of sex hormone receptors in diverse cell types. (A) pancreatic islets, (B) mono- and dinucleated hepatocytes, (C) anterior pituitary cells, (D) adipocytes, and (E) skeletal muscle. (TIF) [file pgen.1010003.s002.tif]

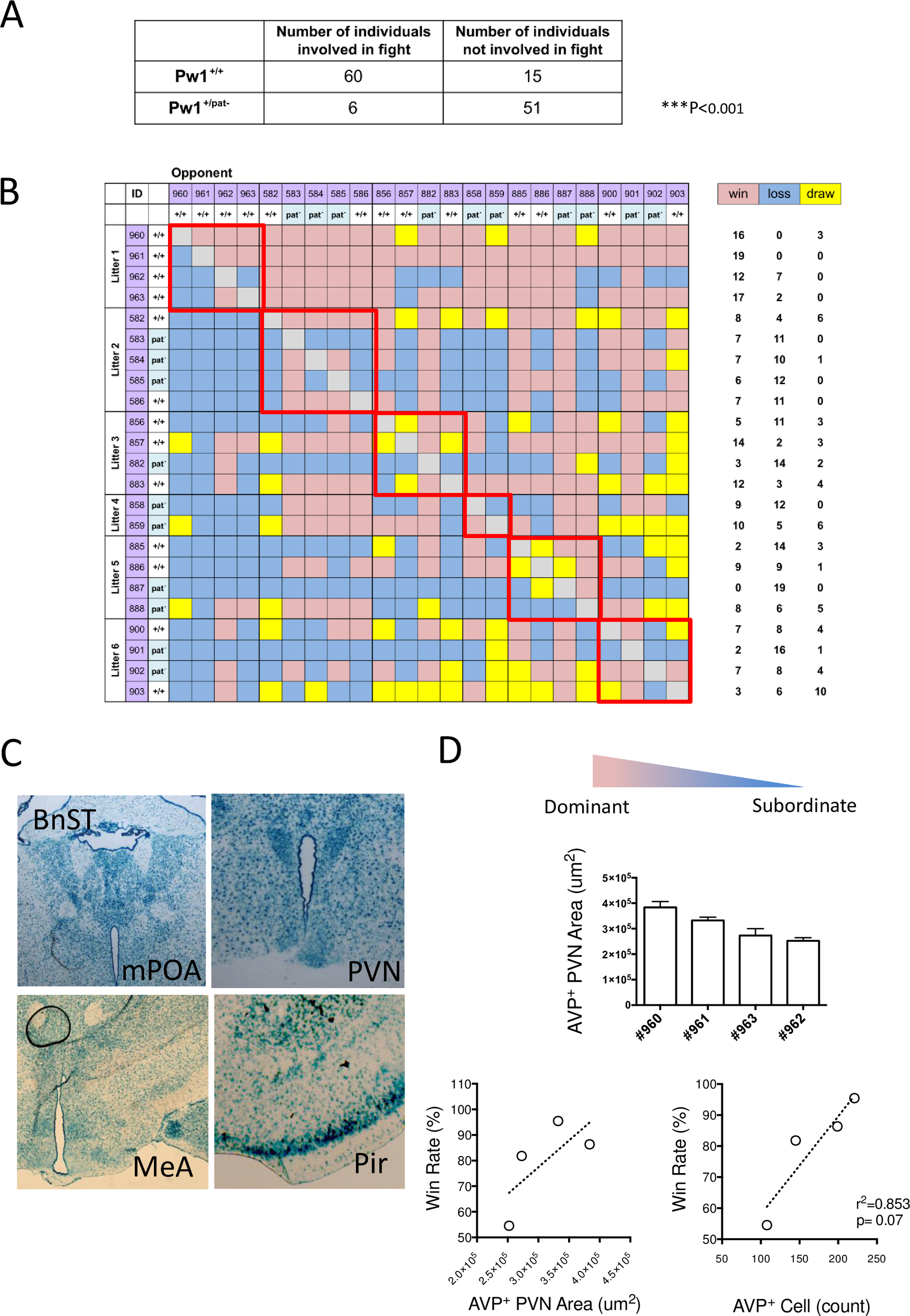

Supplement: S3 Fig — (A) Spontaneous fights among group-housed littermates were monitored per cage and individuals involved in fights were identified. ***, P< 0.001 by Fisher’s exact test. (B) Result of social confrontation tube test in Pw1+/+ (+/+) and Pw1+/pat- (pat-) littermates from six litters. Pw1+/pat- males (n = 10) and Pw1+/+ littermates (n = 13) were subjected for confrontation against each other and their i) intra-litter rank and ii) winning rate against unfamiliar opponents was determined for each animal. Squared in red on the diagonal line show matches within littermates. Mice were derived from C57B6 x Pw1+/p- breeding except for Litter 1, which was derived from C57B6 x Pw1+/+ breeding). (C) Coronal sections of Pw1IRESnLacZ transgenic brain at 2.5 months of age revealing Pw1 reporter gene expression (X-gal staining) in sexually dimorphic brain regions. BnST, the bed nucleus of the stria terminalis; mPOA, medial preoptic area; PVN, paraventricular nucleus of hypothalamus; MeA, medial amygdala; PIR, piriform cortex (x40). (D) Size comparisons of AVP+ PVN area in four Pw1+/+ male siblings from C57B6 x Pw1+/+ breeding (Litter 1). Positive correlation was found between winning rate and AVP+ cells. (TIF) [file pgen.1010003.s003.tif]
