# Supplementary material for: Paternally expressed gene 3 (Pw1/Peg3) promotes sexual dimorphism in metabolism and behavior
Source: PLoS Genet. 2022 Jan 13;18(1):e1010003. doi: 10.1371/journal.pgen.1010003 (PMC8791484; doi:10.1371/journal.pgen.1010003)
Supplement: Attachment — Submitted filename: Review.docx [file pgen.1010003.s004.docx]

Tanaka et al present an interesting manuscript that compares the phenotypes of mice carrying a deletion of the imprinted *Pw1* gene transmitted through the paternal line with wild type (WT) controls. They set out to demonstrate that the *Pw1* deletion has different effects on metabolic and behavioral endpoints in males versus females. The authors suggest that these data provide support for the intralocus sexual conflict model, a proposed model for why genomic imprinting may have evolved.

This is a very interesting story and the manuscript is well written. However, I do have concerns about how some of the experiments were designed, the statistical analyses of some of the data, and the interpretations of the data presented. My biggest concern relates to the assertion that there are sex-specific differences in body metabolism driven by Pw1 deletion, but only a subset of experiments were performed on females, and therefore it is difficult to see how this conclusion can be drawn (see my major point 8 below).

Major concerns

1. In Figure 1A, the authors show data suggesting differences in body weight between WT and Pw1^+/pat-^ mice during postnatal development. Statistical analyses of these differences need to be presented.
2. Also related to Figure 1A, the authors state in the text that there are differences in birth weight but these are not shown in Fig 1A nor is any statistical analysis presented. The data for birth weight should either be shown with an additional graph (with appropriate statistical analysis) or the weights can be presented in the text of the manuscript (again, with appropriate statistical analyses).
3. Again, statistical analyses need to be performed for the data presented in Fig 1B (left), showing a correlation between body weight and glycemia.
4. It is not clear how the statistical analyses of data in Figure 1B (right) and Figure 1C have been performed. The legend mentions a Mann-Whitney U test but this may only refer to panels D and E. If a Mann-Whitney U test was performed, this is not the appropriate way to analyze these data, since this test is designed for 2 independent groups, not 4. Please re-analyze the data using an appropriate test and provide details in the legend.
5. In Figures 2A and 2B, have the data been analyzed with a Mann-Whitney U test? If so, this again is not the appropriate test and should be changed. Also, are the females (WT versus Pw1^+/pat-^) in Figure 2A not significant? If so, this should be indicated as in Figure 1.
6. The authors state that the “gender (sex) difference in IGF-1 dynamics was attenuated in Pw1^+/pat-^ mice, mainly due to the lack of IGF-1 increase in Pw1^+/pat-^ males”. The authors should provide data and statistics to support this idea; as far as I can tell, the idea that this is mainly due to IGF-1 increase in Pw1^+/pat-^ males is based on eyeballing the data in two separate graphs.
7. In Figure 2G, the authors show that liver size and hepatic lipid deposition are increased in Pw1^+/pat-^ males compared to controls at 6 months of age, and state that these “are significantly higher”. However, no statistical comparisons of lipid deposition (oil red O) staining have been performed to support this claim. The oil red O staining should be quantified across multiple individual mice using imaging software or an independent biochemical measurement of hepatic lipid should be performed and analyzed with appropriate statistics.
8. The authors claim that: “Our observations suggest that *Pw1* deficient males develop a gender-specific global shift of body metabolism towards accelerated adiposity, diabetic-like insulin resistance, and fatty liver in adult life”. This claim cannot be made based on the data presented since only males were analyzed for hepatic lipid deposition, hepatic gene expression, ITT and OGTT. Even if the circulating levels of insulin and liver mass (expressed as percent of lean mass) are not significantly different between WT and Pw1^+/pat-^ females, the other metabolic parameters could still be affected. To make the claim that this is a sex-specific effect, which is the major interpretation of this manuscript, the authors need to perform the same set of experiments (oil red O staining and quantification, hepatic gene expression, ITT, OGTT) on WT and Pw1^+/pat-^ female mice.
9. Appropriate statistical analyses need to be performed for Figure S3 panel D, litter 1, to claim that PVN area is correlated with social rank.

Minor concerns

1. While the authors refer to ‘sexual dimorphism’ they also use the term gender. The correct term is sex, since gender refers to social behaviors, identities and expressions. All uses of ‘gender’ should be changed to sex.
2. Prader-Willi syndrome is mis-spelled.
3. The authors state: “Consistently, we found that the levels of major lipogenic genes (such as Scd1 and Pparg) were increased in the *Pw1+/pat-* male liver (Fig 2H).” This is phrased to imply that multiple lipogenic genes including Scd1 and Pparg were increased in expression, when in fact it is only those two. Please rephrase to make this statement accurate.
4. I am not an expert on brain architecture. I understand that the AVP+ PVN is larger in WT males than Pw1^+/pat-^ males but the structure of the region looks very different. Are these sections taken from comparable brain regions and if so can the authors account for the broader structural differences beyond the size of the PVN?
5. In Figure 4C, the expression of StAR is not significantly different between the two groups, which is inconsistent with the description in the text.
