# Supplementary material for: Paternally expressed gene 3 (Pw1/Peg3) promotes sexual dimorphism in metabolism and behavior
Source: PLoS Genet. 2022 Jan 13;18(1):e1010003. doi: 10.1371/journal.pgen.1010003 (PMC8791484; doi:10.1371/journal.pgen.1010003)
Supplement: Attachment — Submitted filename: Response to Reviewers v6.docx [file pgen.1010003.s006.docx]

To the editors and reviewers,

First, we apologize for the extended period required to revise the manuscript, but as there were numerous comments, we sought to answer and revise in response to each comment made. To that end, we thank the reviewers for their careful reading of our study which we know requires time and effort. We have incorporated almost all suggestions made by the reviewers in this revision that we think has led to a greatly improved manuscript.

Below we include a point-by-point response to each comment raised by the 3 reviewers and we outline the specific changes made. All the points raised are numbered for reference. We hope that the reviewers will find this revised version suitable for acceptance.

**Reviewer #1:**

1-Statistical approach: The authors have used paired comparisons but they should use ANOVA, with sex and genotype as factors. By using ANOVA, the authors will be able to make statistically supported conclusions regarding sex effects. For example, in Figure 1A this approach will likely show no effect of SEX and no interaction between SEX and GENOTYPE for body weight and loss of Peg3/Pw1 reduces body weight in both males and females. However, in terms of fat mass (Figure 1E & F), it is likely that an ANOVA would show an interaction between SEX and GENOTYPE, whereby loss of Peg3/Pw1 has little effect in females, but makes males more like WT females. Once this is done, the authors will need to update their abstract etc.

# Response: This point was raised by all 3 reviewers. Accordingly, we have re-analyzed our results using two-way ANOVA and multiple comparisons by Tukey’s honest difference test, to compare the four groups (genotype and sex) for all data applicable in this manuscript.

Our conclusions are as follows: During the younger age when the growth and metabolic characteristics are similar between males and females there is no interaction between sex and genotype, and the parameters of WT vs Pw1^+/pat-^ are significantly different in both sexes (P < 0.0001), as exemplified by body weight up to 3.5-week-old in Figure 1A). By contrast, body weight at 4-week of age shows sex difference (P<0.05) without interaction between sex and genotype, and WT v.s. Pw1^+/pat-^ are significantly different in both sexes (P<0.0001). Finally, body weight at 7-week-old revealed significant interaction between sex and genotype (P<0.05), and Pw1^+/pat-^ are significantly smaller (P<0.0001) in both sexes while females are significantly smaller than males (P<0.0001). Multiple comparisons revealed all groups are significantly different (P<0.0001), except that there is no difference between WT males and Pw1^+/pat-^ females.

Each analysis is detailed in the text and in the figure legends.

2-As an additional point, the authors keep switching comparisons between separating males and females, and Peg3/Pw1 and WT (e.g. Fig 1 D v E; Fig 2 C v G). They should be consistent in their comparisons which could also be resolved with the suggested statistical approach.

Response: To be consistent in the comparison between groups, we integrated four groups of genotype and sex in single panels whenever applicable. This improved the visual presentation of difference between males and females more conclusively; thereby ascertaining phenotypes of Pw1^+/pat-^ are sexually dimorphic.

3- Tube test: There is some confusion regarding which animals are used in the tests to generate scores. The authors mention the use of “adult offspring derived from either Pw1+/+ or Pw1+/pat- males mated with C57/B6 WT females” and Pw1+/+ and Pw1+/pat littermates". Are the authors testing WT and mutant littermates against WT from a fully WT litter bred from Pw1+/+ males or, as indicated in S3, within litter bouts? It is also not clear what is meant by “found that the male progeny derived from Pw1+/pat- males were statistically less aggressive as compared to the ones derived from Pw1+/+ males?” The sentence implies that both mutant and WT males generated from Pw1+/pat- males are less aggressive than WT males from Pw1+/+ males? But there is no data on animals derived from Pw1+/+ males. A true test of social dominance takes into account rank within home cages, which means animals must be tested against littermates, and rank order determined. Sentences should be modified in titles and elsewhere to reflect this.

Another point is that litter 1 is all WT and litter 4 has 2 mutant Peg3 males and no WT. If data from these two litters are excluded, are the findings still significant? Ideally for this test, litters should have mixed genotype in order for rank to be established in the context of genotype.

A third point is that the authors report that animals were initially housed together and then socially isolated before testing, as was done for the Garfield paper cited. There is a second paper on this model where the social group was maintained with no evidence of a social dominance phenotype (Rienecker et al., GBB 2020). This section and the M&M need to be more clearly written and the conclusions reconsidered.

Response: We appreciate these suggestions. The primary purpose of this section was to demonstrate that the paternally expressed gene Pw1/Peg3 regulates masculinization not only in metabolism but also in behavior, the two major contexts in which genomic imprinting is predominantly implicated in mammals, and to elucidate a possible underlying link. Our data point to a role for Peg3 in mediating the establishment of social hierarchy that is predominantly manifested by males.

We separate the data of tube tests in two contexts. The first test was performed to see if the Pw1 is involved in establishing hierarchy within litter using group-housed littermates containing two genotypes. This revealed the social dominant pattern that WT individuals often dominate the siblings and established in higher rank in a given cage at 10-month of age (***P<0.001).

Second test was in a “stranger”-encounter context as performed by Garfield et. al. [1] in which animals were tested with unfamiliar opponents from multiple cages with mixed genotypes, demonstrating that the WT males have statistically less chance to lose in the tube test (**P<0.01). The M&M and the interpretation of the results are now revised in the manuscript.

It is interesting to see the different results in the Grb10 KO models, both of which nonetheless demonstrated the involvement of this imprinted gene in social behaviors. One unsolved question observed by us [2] and others is that the certain phenotypes (such as maternal behavior) in the Peg3 knockout models are often variable and elusive in different genetic background, as well as in different generations after the loss of Pw1, and its underlying mechanisms are not yet elucidated. The contradicting results in Grb10 KO models that was used by Garfield in 2011 and in the second study by Rienecker in 2020 [3] may indicate transgenerational epigenetic differences of the mice analyzed.

4-In the introduction, it would be helpful if the authors provided more explicit details on what behavioural and metabolic phenotypes have been reported in response to loss of Peg3 in their and other models.

Response: We have updated the introduction with detailed descriptions of phenotypes already reported in different Peg3 mutant models, including maternal behavior, male sexual behavior as well as delayed onset of female sexual maturation, all of which are under the sex hormone regulation. We also compared previous reports related to sexually dimorphic outcomes in response to loss of Peg3/Pw1 [4] [5] in our discussion.

**Reviewer #2:**

5- In Figure 1A, the authors show data suggesting differences in body weight between WT and Pw1^+/pat-^ mice during postnatal development. Statistical analyses of these differences need to be presented.

Response: As indicated in our response to Reviewer 1 (please see above), we re-analyzed our data with using two-way ANOVA and multiple comparisons by Tukey’s honest difference test. The difference in growth between WT and Pw1^+/pat-^ is highly significant in all age (***p < 0.0001).

6- Also related to Figure 1A, the authors state in the text that there are differences in birth weight but these are not shown in Fig 1A nor is any statistical analysis presented. The data for birth weight should either be shown with an additional graph (with appropriate statistical analysis) or the weights can be presented in the text of the manuscript (again, with appropriate statistical analyses).

Response: The preceding study conducted on the same model in our laboratory had demonstrated that the birth weight of newborn (P0) is reduced in Pw1^+/pat-^ mice (Correra 2018). We have now modified the text of the manuscript and included the appropriate citation.

7- Again, statistical analyses need to be performed for the data presented in Fig 1B (left), showing a correlation between body weight and glycemia.

It is not clear how the statistical analyses of data in Figure 1B (right) and Figure 1C have been performed. The legend mentions a Mann-Whitney U test but this may only refer to panels D and E. If a Mann-Whitney U test was performed, this is not the appropriate way to analyze these data, since this test is designed for 2 independent groups, not 4. Please re-analyze the data using an appropriate test and provide details in the legend.

Response: We performed linear regression and observed a positive correlation between glycemia and body weight regardless of genotypes (r=0.687, P<0.001).

8- In Figures 2A and 2B, have the data been analyzed with a Mann-Whitney U test? If so, this again is not the appropriate test and should be changed. Also, are the females (WT versus Pw1^+/pat-^) in Figure 2A not significant? If so, this should be indicated as in Figure 1.

Response: We have re-analyzed our data using Two-way ANOVA and updated text and figure legends (Please see question #1). In Fig 2A specifically, two-way ANOVA improved statistical significance, and the IGF-1 level at 3 weeks of age are significantly reduced in Pw1^+/pat-^ (**, P<0.01), with no interaction between sex and genotype.

9-The authors state that the “gender (sex) difference in IGF-1 dynamics was attenuated in Pw1^+/pat-^ mice, mainly due to the lack of IGF-1 increase in Pw1^+/pat-^ males”. The authors should provide data and statistics to support this idea; as far as I can tell, the idea that this is mainly due to IGF-1 increase in Pw1^+/pat-^ males is based on eyeballing the data in two separate graphs.

Response: We have combined the two graphs into one panel and re-analyzed the four groups using two-way ANOVA with multiple comparisons test.

At three weeks of age, there is a significant decrease of IGF-I levels in Pw1^+/pat-^ for both sexes (P<0.01). At five weeks of age, IGF-1 levels are significantly lower in females (WT and Pw1^+/pat-^) as compared to WT males (P<0.001), whereas no differences were demonstrated between Pw1^+/pat-^ males and females. Hence, our conclusion is that the sex difference in IGF-1 level is statistically reduced in Pw1^+/pat-^ genotype.

10- In Figure 2G, the authors show that liver size and hepatic lipid deposition are increased in Pw1^+/pat-^ males compared to controls at 6 months of age, and state that these “are significantly higher”. However, no statistical comparisons of lipid deposition (oil red O) staining have been performed to support this claim. The oil red O staining should be quantified across multiple individual mice using imaging software or an independent biochemical measurement of hepatic lipid should be performed and analyzed with appropriate statistics.

The authors claim that: “Our observations suggest that *Pw1* deficient males develop a gender-specific global shift of body metabolism towards accelerated adiposity, diabetic-like insulin resistance, and fatty liver in adult life”. This claim cannot be made based on the data presented since only males were analyzed for hepatic lipid deposition, hepatic gene expression, ITT and OGTT. Even if the circulating levels of insulin and liver mass (expressed as percent of lean mass) are not significantly different between WT and Pw1^+/pat-^ females, the other metabolic parameters could still be affected. To make the claim that this is a sex-specific effect, which is the major interpretation of this manuscript, the authors need to perform the same set of experiments (oil red O staining and quantification, hepatic gene expression, ITT, OGTT) on WT and Pw1^+/pat-^ female mice.

Response: To answer these comments, we performed additional experiments on our samples from both sexes, which now provide a more complete data set. We have undertaken gene expression and histological analyses on female samples from the same litters to ascertain sex-dependent hepatic phenotypes, and demonstrate further that there is a significant difference in i) hepatic gene expression between males and females (as reported by [6], and ii) adipogenic Pparg2. This difference is specifically elevated in steatotic livers, but significantly lower in females while it is increased only in livers of Pw1^+/pat-^ males (Fig 2G).

Oil red staining and quantification also performed on the liver of both sexes revealed significant increase in the number and size of lipid droplet in Pw1^+/pat-^ . However, the magnitude is severe in males (presented in Fig 2H and Fig S2C).

In general, we aimed to demonstrated that the phenotypic consequences of Pw1 loss of function *in vivo* is significantly different between males and females, but as stated in our discussion, females also exhibit metabolic phenotypes, to a lesser extent, at different time points. We interpret that this is due to differential dependencies on sex steroid hormones rather than an absence of a metabolic phenotypes in females. We have modified our phrasing, in the revised manuscript, from sex-specific to sex-dependent.

11- Appropriate statistical analyses need to be performed for Figure S3 panel D, litter 1, to claim that PVN area is correlated with social rank.

Response: We greatly appreciate this suggestion. Accordingly, we re-examined our result and included additional findings and conclusion in this section. In the litter 1 in particular, the AVP+ cell count and the winning rate in encounter to strangers revealed a positive correlation (R^2^=0,853, p=0,07).

Our data shows that *Pw1* influence vasopressinergic neurons and architecture of the PVN which correlated with the social behavior in males (Fig 3). Further investigations are now needed to explore the exact role of *Pw1* in PVN AVP^+^ neurons, but we believe that it is beyond the scope of the present study.

Pw1/Peg3 is shown to localize in oxytocinergic and vasopressinergic neurons, the two endocrine systems that mediate mammalian social behavior. The previous report on the *Pw1* deficient model described that paternal knockout females display reduced maternal care concomitant with reduced number of oxytocinergic neurons. By contrast, we demonstrate here that Pw1KO males manifest reduced social aggression, another acquired behavior predominantly expressed by males. We hypothesized that *Pw1* is involved in the postnatal sculpturing of specific neurons whose expansion and function depend on sex hormone pathways.

12- While the authors refer to ‘sexual dimorphism’ they also use the term gender. The correct term is sex, since gender refers to social behaviors, identities and expressions. All uses of ‘gender’ should be changed to sex.

Response: We have changed the word gender by sex throughout the revised manuscript.

13- The authors state: “Consistently, we found that the levels of major lipogenic genes (such as Scd1 and Pparg) were increased in the *Pw1+/pat-* male liver (Fig 2H).” This is phrased to imply that multiple lipogenic genes including Scd1 and Pparg were increased in expression, when in fact it is only those two. Please rephrase to make this statement accurate.

Response: We have now updated this section and provide additional results including female data.

14-I am not an expert on brain architecture. I understand that the AVP+ PVN is larger in WT males than Pw1^+/pat-^ males but the structure of the region looks very different. Are these sections taken from comparable brain regions and if so can the authors account for the broader structural differences beyond the size of the PVN?

Response: We examined the entire brains of males with measured behavioral characteristics and analyzed comparable regions consist of 13-16 coronal cross-sections. The brain section images were collected from the same brain coordinates (Bregma: between **-0.70 to -0.94 mm). Typically the AVP^+^ PVN structure spans in four sections in each brain, and we analysed the comparable images for structure analysis.**

Apart from sexually dimorphic regions, we noticed that brain sections from Pw1KO were macroscopically different from WT brains, such as expanded ventricular zone, which may explain the apparent differences of the brain structure.

Since Pw1 and estrogen receptor are also highly expressed in the choroid plexus where the cerebrospinal fluid is produced, it is conceivable that the Pw1 deficiency may have affected the function of this cell type.

15-In Figure 4C, the expression of StAR is not significantly different between the two groups, which is inconsistent with the description in the text.

Response: We have now corrected this error in the revised manuscript.

**Reviewer #3:**

16-It is difficult to determine to what extent the growth retardation in Pat mice is due to early growth deficit, or the combination of this and reduced postnatal growth trajectory. The data in Fig1A should be also plotted as % wild-type (WT) weight, or and/growth rates should be calculated. In this way, deceleration of growth associated with Pw1/Peg3 deficit can be easily visualised and the timing pinpointed.

Response: An earlier published study by our group was conducted on the same model in our laboratory, which had demonstrated that the birth weight of newborns is reduced in Pw1^+/pat-^ males [7]. The growth rate calculated by for both males and females was previously shown in [2]. Here we provide data at the exact stage of sexual dimorphism during postnatal growth (Figure 1A).

17-In Fig1B body weight and glycemia are said to be correlated, yet no statistical value is stated. R or R-squared values as well as p-values should be reported.

Response: We have now updated Figure 1B and provided the corresponding figure legend in the revised manuscript.

18-Food consumption is stated to be lower in mutant mice, yet this is expected if body mass is reduced. These data should be normalised to total body mass and lean body mass to determine if the mutant animals eat a disproportional amount, which would be indicative in a change in central metabolic homeostasis.

Response: We have now updated in the Figure 1C (right). Our revised conclusion is that the sexual dimorphism in food consumption is reduced and the Pw1KO males exhibit feeding behavior comparable to female littermates.

19-The authors claim that because the Pat males resemble the WT females in size, there is therefore an absence of sexual dimorphism in the absence of Pw1/Peg3. This is faulty logic. In terms of the early life growth phenotype the males and females respond similarly to Pw1/Peg3 deletion (i.e., both are growth restricted to a similar extent, as is the body composition prior to 3 months).

Response: We agree with this point, however, we do not claim that the Pw1^+/pat-^ mice lose sexual dimorphism, rather, we show that *Pw1^+/pat-^* mice display a reduced sexual dimorphism at multiple levels during postnatal development. Based on previous studies and the data we provide here in this study, we conclude that the absence of paternal *Pw1* function affects both prenatal and postnatal growth (and is presented in the discussion). We have modified our discussion in the manuscript accordingly.

20-These data are obscured by the presentation in Figure 1D. These data should be shown as in E, with males and females on separate axes, allowing comparison between WT and Pat animals over time. Indeed, Fig1E clearly shows that both males and females have reduced lean mass to a similar degree.

Response: To keep consistent in our comparison of four groups of animals (genotype and sex), we present each result in a single panel (Fig 1D). This makes it clearer that both males and females have reduced lean mass but the degree is more marked in males, as secondary sexual maturation progresses. Hence, while we agree that both males and females have reduced lean mass, it is not to the same degree in adulthood between the sexes. The two-way ANOVA test revealed no effect of sex to lean mass at 1-month-old, after which the influence of sex became significant (****p<0.0001) and interaction between sex and genotype increased (**p<0.01).

21-Further to this, the puberty-associated lean mass does not appear to be affected in the Pat males – compare the gradient of the lean mass increase in Fig1E between genotypes, it is identical. I would rather interpret this to indicate that the developmental deficit in Pw1/Peg3 has caused a reduction in muscle mass (as previously reported by this group and others), and that is maintained into adulthood.

Response: We agree with this interpretation that the reduced body size *in utero* is maintained into adulthood (see above). However, after 3.5-weeks of age, the sexually dimorphic changes appears in the body size and the Pw1^+/p-^ males displayed a further reduction in lean mass (25%), whereas female counterpart showed only 15%, clearly indicating that Pw1 loss of function effects on lean mass is different in males and females. This may account for an additional impact of Pw1 loss of function in males at and after the time point specific to secondary sexual maturation.

Our conclusion is that the Pw1 loss of function reduces body size pre- and postnataly, and masculinization is reduced at multiple levels through multiple pathways. We apologize for the lack of clarity in the original version of the manuscript. We have now modified the text of the revised manuscript to emphasize this point.

22-Statistics applied to repeated measures of body mass is inappropriate. Each animal at a different timepoint, nor fat and lean mass are independent variables, therefore pairwise statistics without multiple comparison correction are not appropriate. Consequently the authors should have performed a 2-Way ANOVA for each sex comparing the effects of time and genotype on lean and fat mass.

Response: We have re-analyzed all the data using 2-way ANOVA, as mentioned in question #1 (see above).

23-Link to IGF1/GH axis: The authors report the interesting finding that IGF1 levels are reduced in 3-week old Pat mice. They attribute this to an alteration in the pituitary GH axis since in males pituitary Gh mRNA levels are reduced. This is an interesting finding, but there are some discrepancies.

Growth retardation is evident from birth, yet in rodents GH does not drive hepatic IGF1 secretion until around the second postnatal week.

The reduction in IGF1 is seen in both sexes (though the female data is underpowered) and is correlated with body size in both sexes, yet pituitary Gh and hepatic Igf1 mRNA levels are only different in males, what is the explanation for this?

Response: Since the hepatic contribution to circulating IGF-I level is shown to be minimal in the postnatal growth, hepatic gene expression of single time point may not reflect the overall activity of IGF-I/GH axis. IGF1/GH axis is dynamically changing during this period and the males and females have very different regulation of IGF-I activity during this period, therefore we might not have been able to capture the impact of Pw1 loss of function in the time frame we analyzed. Nonetheless, the result does not contradict to our conclusion that the Pw1 loss of function affects differently between males and females, and the reduced GH/IGF-I activity is more significant in male Pw1^+/pat-^.

We have analyzed the hepatic Igf1 mRNA expression at 2-week-old and we did not see any difference either between genotype or between sex.

24-Regarding Figure 2C the authors state that there is sexually dimorphic behaviour regarding the male-specific rise in IGF1 post-puberty (which they say does not happen in Pat males). This way of showing the data distorts the picture. Clearly Pats of both sexes have an early IGF1 deficit (~200ng/mL vs ~350ng/mL) which is mostly normalised in the adults of both sexes. The data should be shown with sexes separated and WT and Pat on the same graph.

Response: We have changed the presentation in the figures and performed multiple comparisons at different age. The results are now detailed in the text of the revised manuscript.

Our results did not show that Pw1^+/pat-^ is pathologically defective in growth and metabolisms, but rather, their growth is delayed as compared to WT littermates, and often resemble female counterparts.

25-Is the data in Fig2D from fasted or free-fed animals? The error bar appears to be missing for the 3mo pat females. What is the n for these data?

Response: We have corrected errors and included additional information in the corresponding text and legend of the revised manuscript. Insulin is measured on random-fed as well as fasting animals, except for ITT and OGTT. While reduced glucose and insulin levels were consistently observed in the random-fed Pw1^+/p-^ males during growing phase, fasting animal did not show discernible differences between genotypes.

The analyses presented in Fig2D were performed in n = 6-10 for males, n= 4-8 for females.

26-Fig2G, the paper text says the animals are 6 months old, the figure legend say 10 mo, which is it?

Response: We apologize for this mistake. We have now corrected the errors and included additional information in the corresponding text and legend of the revised manuscript. Age-dependent fatty liver was macroscopically evident from 6 to 10 months old. We further performed steatotic liver analysis using Oil red O staining and digital quantification on the frozen samples collected at 8 months old (n=4-6 each) and presented in Fig. 2H and Fig. S1C.

27-What is the n for Fig2H?

Response: We have now corrected errors and included additional information in the corresponding text and legend of the revised manuscript. Fig2H has been further updated with samples from both males and females and presented in Fig2G (right). n=11 and 15 for male genotypes and n=6 each for females genotypes.

28-In conclusion, I do not feel that the data presented in this paper supports a conclusion that there is sexual dimorphism in lean growth phenotype of Pat animals. Therefore, the later association of this phenotype with masculinising hormones cannot be upheld.

Response: While we have addressed this point above (#17), we would emphasize that we and others showed previously [8] [7] that there is a reduction in body size during prenatal development, the differences in growth between WT and Pw1^+/p-^ littermates become more significant in males during postnatal development and the degree of pathological manifestation is more severe in males. It seems likely, as we propose, that there is an involvement of sex hormones and that while the basis of these differences may be the same, the outcomes are not. Our view is that the Pw1 loss of function affects sex hormone dependent physiology pre- and postnatally, and they are not mutually exclusive. We apologize for the lack of clarity. Accordingly, we have now changed the text in the revised version of our manuscript.

29-While the observation of reduced IGF1 in Pats is interesting, it is present in both sexes and cannot be entirely explained by alteration of the pituitary GH axis.

However, there is convincing evidence of male-specific adipose weight gain in the mutant animal in later life, and this is associated with the expected glucose intolerance, beta cell expansion and hyperglycaemia. While interesting, the authors are not able to provide an explanation for this at the molecular level. Moreover, such changes in body composition and metabolism have previously been reported by the Keverne/Curley group in an independent Pw1/Peg3 deletion model.

Response: We respectfully disagree with the reviewer. The previous study by Keverne/Curley group demonstrated metabolic changes in male Pw1^+/pat-^ and did not show any data on female counterparts. Therefore, while we are confirming a consistent changes in metabolism upon paternal Pw1/Peg3 loss in males, the significance and the interpretation are completely different in the light of sexual differentiation. Moreover, the biochemical analysis in the previous study showed serum biochemistry of mice at 6-month of age when the growth and sexual maturation is completed, while the only hormone that are significantly different between genotypes are leptin levels resulting from the increased fat mass in male Pw1^+/pat-^. We demonstrate here that Pw1/Peg3 loss of function affected multiple stages of early life that proceeded to the physiological changes in later adulthood.

30-Behavioural phenotyping and localisation of PW1/PEG3 in the adult male brain.

What statistics were performed on the behavioural data? This should be stated.

Response: The section of behavioral phenotype and methodology has been re-examined and updated in the manuscript, in response to the question #2 (Reviewer 1).

31-Expression of PEG3 in the AVP neurons of the PVN has been previously reported: <https://pubmed.ncbi.nlm.nih.gov/12399444/>

Response: We agree with the reviewer. Consistent with the publication mentioned above, we confirmed that Pw1/Peg3 reporter gene is expressed in both OXT and AVP neurons. However, we show here that in the female Pw1^+/p-^ brain, oxytocinergic architecture was found to be hypomorphic [9], concomitant with an insufficient maternal behavior. In the current study, we focused on the male specific behavior that is shown to be regulated by the vasopressinergic pathway. While further behavioral studies may be warranted, we have confirmed a statistically significant difference in social dominant test and that global vasopressinergic architectures were also altered in the male Pw1^+/p-^. Therefore, we have confirmed a previous study by others and have extended upon those results.

32-In Fig4A how many animals were assayed, this is not stated? Also, the stats is inappropriate, it should be analysed by One-Way ANOVA since the individuals at multiple timepoints are not independent. The error bar for the Pat at 9 weeks is missing.

Response: We followed two genotypes (WT vs KO n=7 and n=5) within the littermates from 3 litters. Error bar is presented in the figure but there is very small variance in KO.

ANOVA test revealed a significant effect of age and multiple comparisons of four time points showed week 9 has a significant difference from week 1 & 2 in WT, but not in in KO, suggesting the testosterone surge is delayed in KO males.

33-The ovelap between PW1/PEG3 expression and AR in the gonads is minimal – would this be sufficient to account for the reduction in testosterone observed in Fig4B?

Response: We show that PW1/PEG3 is expressed in different cells types that are sex-hormone dependent. However, we did not identify if the testosterone secretion is defective in Pw1^+/pat-^ Leidig cells *in vitro*. Unfortunately, it is difficulty is to maintain the imprinting status of Pw1 in cell culture conditions.

34-Would a transient reduction of testosterone observed be sufficient to account for the metabolic phenotype? The critical causal experiment would be to replace testosterone levels in mutant mice at this age, and see if the metabolic and behavioural phenotypes were reversed. In conclusion, the observed transient reduction in testosterone levels, and association of PW1/PEG3 expression with sex hormone-responsive cell populations is a very interesting finding. However, at this stage the work appears preliminary with not much causal link to phenotype. A hormone replacement/conditional deletion experiment would be required to elevate this work.

Response: We thank the reviewer for these pertinent comments and suggestions, but really are a foundation for another study beyond the scope of this manuscript. Indeed, we focused our scope of investigation here on the *in vivo* consequence(s) of Pw1/Peg3 loss of function in order to identify specific periods of postnatal life. To our knowledge, this is the first study to demonstrate comparison of the postnatal development and metabolism of males and females head-to-head in relation to an imprinted gene. The molecular involvement of Pw1/Peg3 in sex steroid pathways in various co-expressing cells is of our utmost interest, and has been studied *in vitro*. We are presently pursuing the very topics that the reviewer has raised, but again, we feel that the present study provides the groundwork for future work of this nature.

Hormone replacement by GH and testosterone is certainly an important consideration, as it is in practice for patients with PWS. Our prediction is that it will ameliorate some of the manifestations observed in Pw1^+/pat-^, but since the onset of phenotypes starts prenatally and continue to evolve throughout life, the effects are likely to be incomplete. As one example, we are examining the potential to rescue the muscle phenotype in mutant mice using a pharmacological approach that has been shown to ameliorate testosterone deficiency by increasing muscle mass and decreasing fat mass. We observed both WT and Pw1^+/pat-^ responded to the treatment with significant increase of lean mass at the similar levels, which may indicate that there is no defect in muscle growth and development at peripheral levels in Pw1^+/pat-^ animals. Clearly, unraveling this complex and ‘whole body’ phenotype will require further study.

1.	Garfield AS, Cowley M, Smith FM, Moorwood K, Stewart-Cox JE, Gilroy K, et al. Distinct physiological and behavioural functions for parental alleles of imprinted Grb10. Nature. 2011;469(7331):534-8. doi: 10.1038/nature09651. PubMed PMID: 21270893; PubMed Central PMCID: PMCPMC3031026.

2.	Denizot AL, Besson V, Correra RM, Mazzola A, Lopes I, Courbard JR, et al. A Novel Mutant Allele of Pw1/Peg3 Does Not Affect Maternal Behavior or Nursing Behavior. PLoS Genet. 2016;12(5):e1006053. doi: 10.1371/journal.pgen.1006053. PubMed PMID: 27187722; PubMed Central PMCID: PMCPMC4871489.

3.	Rienecker KDA, Chavasse AT, Moorwood K, Ward A, Isles AR. Detailed analysis of paternal knockout Grb10 mice suggests effects on stability of social behavior, rather than social dominance. Genes, brain, and behavior. 2020;19(1):e12571. Epub 2019/04/02. doi: 10.1111/gbb.12571. PubMed PMID: 30932322; PubMed Central PMCID: PMCPMC7050506.

4.	Kim J, Frey WD, He H, Kim H, Ekram MB, Bakshi A, et al. Peg3 mutational effects on reproduction and placenta-specific gene families. PLoS One. 2013;8(12):e83359. Epub 2014/01/07. doi: 10.1371/journal.pone.0083359. PubMed PMID: 24391757; PubMed Central PMCID: PMCPMC3877027.

5.	Tunster SJ, Boqué-Sastre R, McNamara GI, Hunter SM, Creeth HDJ, John RM. Peg3 Deficiency Results in Sexually Dimorphic Losses and Gains in the Normal Repertoire of Placental Hormones. Frontiers in cell and developmental biology. 2018;6:123. Epub 2018/10/16. doi: 10.3389/fcell.2018.00123. PubMed PMID: 30320110; PubMed Central PMCID: PMCPMC6170603.

6.	Rando G, Wahli W. Sex differences in nuclear receptor-regulated liver metabolic pathways. Biochim Biophys Acta. 2011;1812(8):964-73. doi: 10.1016/j.bbadis.2010.12.023. PubMed PMID: 21211563.

7.	Correra RM, Ollitrault D, Valente M, Mazzola A, Adalsteinsson BT, Ferguson-Smith AC, et al. The imprinted gene Pw1/Peg3 regulates skeletal muscle growth, satellite cell metabolic state, and self-renewal. Sci Rep. 2018;8(1):14649. doi: 10.1038/s41598-018-32941-x. PubMed PMID: 30279563; PubMed Central PMCID: PMCPMC6168517.

8.	Curley JP, Pinnock SB, Dickson SL, Thresher R, Miyoshi N, Surani MA, et al. Increased body fat in mice with a targeted mutation of the paternally expressed imprinted gene Peg3. FASEB J. 2005;19(10):1302-4. doi: 10.1096/fj.04-3216fje. PubMed PMID: 15928196.

9.	Li L, Keverne EB, Aparicio SA, Ishino F, Barton SC, Surani MA. Regulation of maternal behavior and offspring growth by paternally expressed Peg3. Science. 1999;284(5412):330-3. PubMed PMID: 10195900.
