# Supplementary material for: Paternally expressed gene 3 (Pw1/Peg3) promotes sexual dimorphism in metabolism and behavior
Source: PLoS Genet. 2022 Jan 13;18(1):e1010003. doi: 10.1371/journal.pgen.1010003 (PMC8791484; doi:10.1371/journal.pgen.1010003)
Supplement: Attachment — Submitted filename: Responses to the reviewers 2nd.docx [file pgen.1010003.s007.docx]

To the editor,

Thank you for your patience during this revision process. We have addressed the remaining comments from the reviewers and thank them for their careful reading of this study that we agree has led to a significantly improved manuscript. We hope that you will find this study acceptable for publication. Below, please find a point-by-point response to each comment raised. In addition to the changes indicated below, we note that we have also added three more names to the acknowledgement as well as minor adjustments to font size and size of panels in the final figures to increase legibility.

**Reviewer #1:**

*The authors have undertaken considerable work to address comments made by reviewers, which significantly improve the understanding of the manuscript. As a minor point, while tests for aggression have predominantly been developed on male rodents, non-pregnant females can exhibit aggression (Oliveira 2021). Moreover, there is evidence of increased aggression in lactating Peg3 mutant females (Champagne 2009). The authors have not tested aggression in females in this study.*

*The title of the section related to tube test should be amended.*

*From*

*Paternal Pw1 deficiency reduces male-specific aggressive behaviors and social dominance behavior.*

*To*

*Paternal Pw1 deficiency reduces aggressive behaviors and social dominance.behavior in males.*

Response:

We very much appreciate these suggestions.

Accordingly, we have changed the title of the section in the manuscript.

Current study focused on masculinization of behavior in males, but the female social behaviors were also affected, as we equally observed a reduced social dominance in Pw1 mutant females. Our female data are limited, however, and further clarification is necessary at this point.

Since the vasopressinergic & oxytocinergic neurons are Pw1 expressing cells, male and females are expected to reveal resulting phenotypes in different consequences.

**Reviewer #2:**

*The authors have done a good job responding to my concerns and the manuscript is significantly improved. I suggest that the manuscript requires 'minor revisions' only because there are a few errors throughout the text.*

*Some examples include "Body weight at 7-week-old revealed..." (this should read "Body weight at 7 weeks of age revealed...") and "Sex differences emerges..." (this should read "Sex differences emerge..."). I suggest the authors go through the manuscript one more time to correct these and other grammatical and sentence structure issues.*

Response:

We carefully revised the entire manuscript and corrected grammatical and typing errors including those mentioned above. We also converted English (UK) to English (US).

Appearance order of reference was modified without changing the content and context.

On behalf of the authors

David Sassoon
